# Patterns of Distant Organ Metastasis and Risk Factors in Rectal Adenocarcinoma Patients: A Retrospective Cohort Analysis

**DOI:** 10.7759/cureus.90095

**Published:** 2025-08-14

**Authors:** Mohammad Ebad Ur Rehman, Faizan Fazal, Arham A Khokhar, Saad Tahir, Usama Tanveer, Hamza Yunus, Ahmad Iftikhar, Beenish Sabir, Areesha Abid, Fatima Faraz, Bilal Haider Malik

**Affiliations:** 1 Department of Medicine, Holy Family Hospital, Rawalpindi, PAK; 2 Trauma and Orthopaedics Surgery, Sherwood Forest Hospitals NHS Foundation Trust, Sutton-in-Ashfield, GBR; 3 Internal Medicine, Hayatabad Medical Complex Peshawar, Peshawar, PAK; 4 Internal Medicine, University of Arizona, Tucson, USA; 5 Medical School, Rawalpindi Medical University, Rawalpindi, PAK; 6 Dermatology, Betsi Cadwaladr University Health Board, Wrexham, GBR

**Keywords:** adenocarcinoma, cancer metastasis, rectal cancer, retrospective cohort, risk factors

## Abstract

Background

In this study, we aimed to analyze the metastatic patterns of rectal adenocarcinoma (RAC) to specific organs, assessing the incidence, associated risk factors, and prognostic implications of metastasis.

Methodology

We conducted a retrospective cohort study utilizing data from the Surveillance, Epidemiology, and End Results database, focusing on RAC patients diagnosed between 2010 and 2018. Analyses were performed to evaluate overall survival (OS) and cancer-specific survival (CSS).

Results

We included 13,564 patients with RAC. Significant risk factors for metastasis were age <50 years (odds ratio (OR) = 1.168), grade 4 (OR = 2.119), T4 (OR = 1.866), N2 (OR = 4.520), and patients without surgery (OR = 9.71). The median OS and CSS in metastatic RAC were 24 and 25 months, respectively. Variables significantly associated with decreased OS were old age, high-grade tumors, T4, N2, tumors >200 mm, no surgery, and multiple metastatic sites. The variables significantly associated with reduced CSS were old age, high-grade tumors, T4, N2, tumors larger than 200 mm, no surgery, and multiple metastatic sites.

Conclusions

The liver was the most common organ in which metastasis occurred. Significant risk factors observed in RAC patients for metastasis were age <50 years, grade 4, T4, N2, and patients without surgery. Old age, multiple metastatic sites, high-grade tumors, T4, N2, tumors larger than 200 mm, and no surgery were strongly linked to a lower OS rate.

## Introduction

Adenocarcinoma is a type of cancer that starts in the gland cells that make mucus to lubricate and protect the inside of the colon and rectum. Completely differentiated adenocarcinomas resemble the glandular tissue from where they originated [1]. Rectal cancer, the second most common kind of colorectal cancer (CRC), is the malignant growth of glandular tissue in the rectum. Adenocarcinoma is the most prevalent form of rectal cancer (90%) [2]. Other rare rectal carcinomas include adenosquamous, spindle, squamous, and undifferentiated variants. CRC is the second most common cancer in the world and one of the most important public health issues, necessitating heightened awareness [2].

Gene mutations, including those involving DNA repair processes, oncogenes, and tumor suppressor genes, cause rectal adenocarcinomas (RACs) [3]. Adenocarcinomas developing in the rectum and colon have various diagnostic features, risk factors, and prognosis, considering the separate embryonic origins from the midgut and hindgut into account [4]. The rectum is exposed to a more concentrated environment than the colon, which is in contact with less concentrated, undigested, mucus-coated feces. The differences in pH levels between the rectum and colon may account for a mutation-inducing environment [5].

The management and workup of RAC demands comprehensive knowledge regarding its precise anatomical location. According to the National Cancer Institute consensus panel, the rectum is a 12-cm-long tube beginning from the anal verge, confirmed using solid proctoscopy [6]. Due to the anatomical location of the rectum, which differentiates it from the colon, the narrow and bony pelvic boundaries make surgical resection of the tumor more difficult. The absence of serosa below the peritoneum promotes the development of deeper and more invasive tumors within the perirectal fat, leading to higher rates of locoregional failure [7]. The anatomical differences between the colon and rectum account for rectal cancer risks that are four times higher than colon cancer risks [8]. Rectal carcinomas are further differentiated from colonic carcinomas in terms of mortality and complications. Riihimäki et al. reported that colonic carcinoma patients had a significantly higher short-term mortality compared to rectal carcinoma patients due to a more severe effect of complications [9].

Metastasis refers to the distant development of secondary malignant proliferative sites. The hallmark of cancer that is responsible for the greatest number of cancer-related deaths globally is metastasis to secondary sites [10]. Metastasis is a leading cause of death in colon or rectal cancer, with over 56% of CRC patients passing away due to metastasis [11]. Pretzsch et al. reported the peritoneum and liver as the most common sites of metastasis in patients with CRC [12]. Based on a Swedish cancer database study, rectal cancers tend to spread to the thoracic and nervous systems, but less frequently to the peritoneum [9]. The liver is the most common organ to be metastasized with the worst prognosis in CRC patients [13]. The carcinogenesis, pathology, prognosis, and surgical and treatment plans of colon and rectal cancer are different [14]. There is a need for additional research into the metastatic pattern of RAC and the risk factors that contribute to its development. As a result, studying the pattern of distant organ metastasis in RAC patients should be a primary concern, as massive organ damage can be fatal. Our population-based study aimed to investigate the risk factors associated with distant organ metastasis in RAC patients. To our knowledge, this is the first Surveillance, Epidemiology, and End Results (SEER)-based study to offer a comprehensive analysis of distant organ metastasis specifically in RAC patients. Our findings present a broad perspective on metastatic patterns in RAC, aimed at helping clinicians make more informed and effective decisions.

## Materials and methods

Study design and study sample

This retrospective cohort study included 13,564 patients with diagnosed RAC. Existing data were investigated between exposure and outcome, looking backwards in time.

Data source

The data were retrieved from the SEER database, which is a data registry funded by the National Cancer Institute. The SEER is the largest cancer database that has been made available publicly.

Eligibility criteria

This study only included patients with RAC, confirmed by histopathological reports mentioned in the database. We excluded all patients who had incomplete follow-up and those in whom the rectal tumor was not the primary malignancy.

Outcome variables

We collected information related to Tumor, Node, Metastasis (TNM) staging; gender; age; race; histology; tumor size; grade; surgery status; and metastatic site information. No further ethical clearance was needed after authorization was acquired from the SEER website.

Statistical analysis

Demographic characteristics (gender, age, and monthly income), tumor grading, tumor staging, and surgery status of patients with and without metastasis were analyzed using descriptive statistics. The descriptive statistics (means ± SD or frequencies/percentages) were used appropriately for different variables. The “tumor grading/staging” and “surgery status” were treated as categorical variables. For categorical and continuous data, the Pearson’s chi-square test and the Student’s t-test were employed, respectively. A multivariable logistic regression model was utilized to identify the risk factors of distant organ metastasis to the bone, brain, liver, and lung. Cox proportional hazards regression was undertaken to identify prognostic factors for patients with metastasis. This regression model was used to explore the relationship between the survival time of patients and one or more predictor variables. All analysis was conducted using SPSS version 26, and a p-value of less than 0.05 was regarded as statistically significant.

## Results

Characteristics of patients with or without metastasis

The analysis consisted of 13,564 patients with RAC diagnosed from 2010 to 2018, including 8,042 (59.3%) males and 5,522 (40.7%) females. The patients from stage I to IV were 7.6%, 78.8%, 12.0% and 1.7%, respectively. The database had metastatic site information for the liver, lung, brain, and bone only. Metastasis to any of the sites was reported in 1,559 (11.5%) patients. Liver metastases were more frequently noted (9.6%), followed by the lung (4.1%), bone (0.8%), and brain (0.1%). The incidence of metastasis was comparable in males and females. However, liver metastasis was more common in males (10.2% vs. 8.7%), and brain metastasis was more common in females. African American patients had a greater incidence of metastasis (14.8%), liver metastasis (12.7%), and lung metastasis (5.9%) compared to Caucasians and other races. Metastasis to the liver was significantly more common in patients aged less than 50 years compared to those aged older than 50 years (p < 0.001). Patients with metastasis had significantly larger tumors, higher grade, higher T stage, and higher N stage compared to patients without metastasis. Patients who did not undergo surgery had a higher incidence of metastasis compared to patients who had undergone surgery (38.2% vs. 7.4%). Table 1 summarizes the clinico-pathological characteristics of patients with and without metastasis.

**Table 1:** Patients’ characteristics with and without metastasis compared using the chi-square test and Student’s t-test.

Features	Any metastasis, N (%)	No metastasis, N (%)	P-value	Liver metastasis, N (%)	No liver metastasis, N (%)	P-value	Brain metastasis, N (%)	No brain metastasis, N (%)	P-value	Bone metastasis, N (%)	No bone metastasis, N (%)	P-value	Lung metastasis, N (%)	No lung metastasis, N (%)	P-value
Total	1,559	12,005		1,301	12,263		18	13,546		106	13,458		558	13,006	
Race															
Caucasian	1,196 (11.2%)	9439 (88.8%)	0.001	1,001 (9.4%)	9,634 (90.6%)	0.001	15 (0.1%)	10,620 (99.9%)	0.606	77 (0.7%)	10,558 (99.3%)	0.336	422 (4%)	10,213 (96%)	0.008
African American	169 (14.8%)	974 (85.2%)		145 (12.7%)	998 (87.3%)		2 (0.2%)	1,141 (99.8%)		12 (1%)	1,131 (99%)		67 (5.9%)	1,076 (94.1%)	
Other	194 (10.9%)	1,592 (89.1%)		155 (8.7%)	1631 (91.3%)		1 (0.1%)	1,785 (99.9%)		17 (1%)	1,769 (99%)		69 (3.9%)	1,717 (96.1%)	
Sex															
Male	958 (11.9%)	7,084 (88.1%)	0.065	822 (10.2%)	7,220 (89.8%)	0.003	6 (0.1%)	8,036 (99.9%)	0.025	66 (0.8%)	7,976 (99.2%)	0.531	312 (3.9%)	7,730 (96.1%)	0.097
Female	601 (10.9%)	4,921 (89.1%)		479 (8.7%)	5,043 (91.3%)		12 (0.2%)	5,510 (99.8%)		40 (0.7%)	5,482 (99.3%)		246 (4.5%)	5,276 (95.5%)	
Age															
Less than 50	339 (14.0%)	2,085 (86%)	<0.001	298 (12.3%)	2,126 (87.7%)	<0.001	4 (0.2%)	2,420 (99.8%)	0.784	26 (1.1%)	2,398 (98.9%)	0.193	105 (4.3%)	2,319 (95.7%)	0.416
50–75	941 (11.7%)	7,123 (88.3%)		789 (9.8%)	7,275 (90.2%)		11 (0.1%)	8,053 (99.9%)		59 (0.7%)	8,005 (99.3%)		339 (4.2%)	7,725 (95.8%)	
More than 75	279 (9.1%)	2,797 (90.1%)		214 (7%)	2,862 (93%)		3 (0.1%)	3,073 (99.9%)		21 (0.7%)	3,055 (99.3%)		114 (3.7%)	2,962 (96.3%)	
Grade															
1	64 (6.2%)	963 (93.8%)	<0.001	56 (5.5%)	971 (94.5%)	<0.001	0 (0%)	1,027 (100%)	0.031	1 (0.1%)	1,026 (99.9%)	0.003	25 (2.4%)	1,002 (97.6%)	0.029
2	1194 (11.2%)	9,488 (88.8%)		993 (9.3%)	9,689 (90.7%)		12 (0.1%)	10,670 (99.9%)		80 (0.7%)	10,602 (99.3%)		446 (4.2%)	10,236 (95.8%)	
3	266 (16.4%)	1,355 (83.6%)		223 (13.8%)	1,398 (86.2%)		6 (0.4%)	1,615 (99.6%)		22 (1.4%)	1,599 (98.6%)		76 (4.7%)	1,545 (95.3%)	
4	35 (15%)	199 (85%)		29 (12.4%)	205 (87.6%)		0 (0%)	234 (100%)		3 (1.3%)	231 (98.7%)		11 (4.7%)	223 (95.3%)	
T stage															
1	249 (8.6%)	2633 (91.4%)	<0.001	214 (7.4%)	2668 (92.6%)	<0.001	8 (0.3%)	2,874 (99.7%)	0.04	25 (0.9%)	2,857 (99.1%)	<0.001	98 (3.4%)	2,784 (96.6%)	<0.001
2	75 (3.6%)	2,007 (96.4%)		59 (2.8%)	2,023 (97.2%)		0 (0%)	2,082 (100%)		8 (0.4%)	2,074 (99.6%)		28 (1.3%)	2,054 (98.7%)	
3	860 (12.2%)	6,172 (87.8%)		731 (10.4%)	6,301 (89.6%)		7 (0.1%)	7,025 (99.9%)		43 (0.6%)	6,989 (99.4%)		281 (4%)	6,751 (96%)	
4	375 (23.9%)	1,193 (76.1%)		297 (18.9%)	1,271 (81.1%)		3 (0.2%)	1,565 (99.8%)		30 (1.9%)	1,538 (98.1%)		151 (9.6%)	1,417 (90.4%)	
N															
0	490 (6.5%)	7,041 (93.5%)	<0.001	396 (5.3)	7,135 (94.7)	<0.001	6 (0.1)	7,525 (99.9)	0.002	44 (0.6%)	7,487 (99.4%)	0.014	188 (2.5%)	7,343 (97.5%)	<0.001
1	711 (16.2%)	3,679 (83.8%)		597 (13.6)	3793 (86.4)		5 (0.1)	4,385 (99.9)		46 (1.0%)	4,344 (99.0%)		261 (5.9%)	4,129 (94.1%)	
2	358 (21.8%)	1,285 (78.2%)		308 (18.7)	1,335 (81.3)		7 (0.4)	1636 (99.6)		16 (1.0%)	1,627 (99.0%)		109 (6.6%)	1,534 (93.4%)	
Surgery															
Yes	870 (7.4%)	10,889 (92.6%)	<0.001	736 (6.3%)	11023 (93.7%)	<0.001	10 (0.1%)	11,749 (99.9%)	<0.001	32 (0.3%)	11,727 (99.7%)	<0.001	251 (2.1%)	11,508 (97.9%)	<0.001
No	689 (38.2%)	1,116 (61.8%)		565 (31.3%)	1,240 (68.7%)		8 (0.4%)	1,797 (99.6%)		74 (4.1%)	1,731 (95.9%)		307 (17%)	1,498 (83.0%)	
Size	260.52	187.51	<0.001	476.72	193.69	<0.001	225.11	195.86	0.723	476.72	193.69	<0.001	308.81	191.06	<0.001
Income															
Less than 50,000	234 (13.1%)	1,557 (86.9%)	0.076	193 (10.8%)	1,598 (89.2%)	0.179	8 (0.4%)	1,783 (99.6%)	<0.001	17 (0.9%)	1,774 (99.1%)	0.683	87 (4.9%)	1,704 (95.1%)	0.215
50,000–75,000	791 (11.2%)	6,296 (88.8%)		662 (9.3%)	6,425 (90.7%)		6 (0.1%)	7,081 (99.9%)		53 (0.7%)	7,034 (99.3%)		279 (3.9%)	6,808 (96.1%)	
More than 75,000	534 (11.4%)	4,152 (88.6%)		446 (9.5%)	4,240 (90.5%)		4 (0.1%)	4,682 (99.9%)		36 (0.8%)	4,650 (99.2%)		192 (4.1%)	4,494 (95.9%)	

Risk factors for developing metastasis

We performed multivariate logistic regression and calculated the adjusted odds ratio (aOR) to identify risk factors for developing metastasis in RAC. The risk of metastasis to any site was significantly lower in patients aged 50-75 years (aOR = 0.856, 95% confidence interval (CI) = 0.738-0.992) and patients aged >75 years (aOR = 0.523, 95% CI = 0.432-0.633) compared to patients aged <50 years. The risk of metastasis was significantly higher in grade 2 (aOR = 1.698, 95% CI = 1.283-2.247), grade 3 (aOR = 1.826, 95% CI = 1.338-2.492), and grade 4 (aOR = 2.119, 95% CI = 1.317-3.412) compared to grade 1 cancer. Patients at the T2 stage had a significantly lower risk of metastasis (aOR = 0.445, 95% CI = 0.335-0.591), and those at the T4 stage had a significantly higher risk of metastasis (aOR = 1.866, 95% CI = 1.521-2.289) compared to patients at the T1 stage. Patients at the N1 stage (aOR = 2.511, 95% CI = 2.180-2.893) and N2 stage (aOR = 4.520, 95% CI = 3.786-5.397) had an increased risk of metastasis compared to patients at the N0 stage. Patients who underwent surgery were at decreased risk of metastasis (aOR = 0.103, 95% CI = 0.090-0.118) versus those who did not undergo surgery. Reduced risk of metastasis was reported in patients with an income between 50,000 and 75,000 USD (aOR = 0.818, 95% CI = 0.688-0.972) and those with an income greater than 75,000 USD (aOR = 0.818, 95% CI = 0.679-0.986) compared to patients with an income less than 50,000 USD. Details of these risk factors for distant organ metastasis are presented in Table 2.

**Table 2:** Multivariate logistic regression model for odds of site-specific metastases.

Features	Any metastasis	Liver	Brain	Bone	Lung
	aOR (95% CI)	aOR (95% CI)	aOR (95% CI)	aOR (95% CI)	aOR (95% CI)
Race					
White	1	1	1	1	1
Black	1.039 (0.855-1.263)	1.078 (0.878-1.324)	0.625 (0.133-2.939)	0.972 (0.517-1.829)	1.122 (0.844-1.492)
Other	0.991 (0.826-1.189)	0.925 (0.76-1.127)	0.486 (0.061-3.860)	1.481 (0.843-2.601)	1.049 (0.791-1.392)
Gender					
Male	1	1	1	1	1
Female	0.972 (0.862-1.095)	0.887 (0.78-1.008)	3.041* (1.122-8.242)	0.995 (0.663-1.491)	1.298* (1.083-1.556)
Age					
Less than 50	1	1	1	1	1
50-75	0.856* (0.738-0.992)	0.808* (0.692-0.943)	0.776 (0.238-2.532)	0.640 (0.396-1.035)	1.003 (0.791-1.271)
More than 75	0.523* (0.432-0.633)	0.466* (0.38-0.571)	0.447 (0.093-2.138)	0.454* (0.247-0.937)	0.706* (0.527-0.946)
Tumor grade					
1	1	1	1	1	1
2	1.698* (1.283-2.247)	1.561* (1.162-2.095)	1,598,426.821	7.066 (0.976-51.143)	1.52 (0.995-2.321)
3	1.826* (1.338-2.492)	1.686* (1.215-2.338)	4,038,001.292	8.843* (1.174-66.596)	1.154 (0.712-1.868)
4	2.119* (1.317-3.412)	1.905* (1.148-3.160)	0.912	14.715* (1.484-145.946)	1.7 (0.798-3.621)
T stage					
1	1	1	1	1	1
2	0.445* (0.335-0.591)	0.396* (0.29-0.541)	0	0.816 (0.357-1.869)	0.519* (0.334-0.807)
3	1.070 (0.896-1.277)	1.007 (0.834-1.215)	0.192* (0.058-0.640)	0.776 (0.45-1.341)	1.006 (0.771-1.311)
4	1.866* (1.521-2.289)	1.533* (1.233-1.905)	0.24 (0.054-1.068)	1.495 (0.837-2.67)	1.959* (1.461-2.626)
Nodal status					
0	1	1	1	1	1
1	2.511* (2.180-2.893)	2.514* (2.16-2.925)	1.857 (0.503-6.858)	1.57 (0.999-2.469)	2.207* (1.783-2.732)
2	4.520* (3.786-5.397)	4.6* (3.81-5.553)	9.447* (2.445-36.498)	2.207* (1.166-4.19)	3.371* (2.554-4.451)
Surgery					
No	1	1	1	1	1
Yes	0.103* (0.090-0.118)	0.119* (0.103-0.138)	0.163* (0.058-0.462)	0.074* (0.047-0.116)	0.102* (0.084-0.124)
Tumor size					
<=200 mm	1	1	1	1	1
>200 mm	0.987 (0.843-1.155)	0.972 (0.824-1.148)	0.491 (0.128-1.881)	2.041* (1.335-3.120)	1.157 (0.929-1.441)
Income					
Less than 50,000	1	1	1	1	1
50,000–75,000	0.818* (0.688-0.972)	0.844 (0.702-1.015)	0.17* (0.057-0.513)	0.846 (0.479-1.495)	0.802 (0.617-1.042)
More than 75,000	0.818* (0.679-0.986)	0.848 (0.695-1.035)	0.18* (0.05-0.643)	0.813 (0.439-1.506)	0.813 (0.613-1.079)

Survival in patients with metastasis

**Figure 1:**
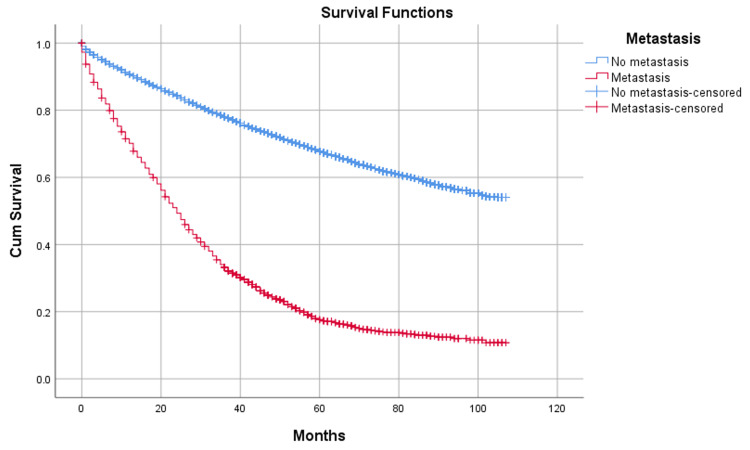
Survival in patients with metastasis.

The median overall survival (OS) was 24 months (95% CI = 22.5-25.5 months) in patients with metastasis and was not reached in patients without metastasis (p < 0.001). The median cancer-specific survival (CSS) was 25 months (95% CI = 23.4-26.6 months) in patients with metastasis and was not reached in patients without metastasis. OS and CSS both decreased significantly with an increase in the number of metastatic sites. The median OS of one, two, and three sites of metastasis were 27, 15, and 8 months, respectively (p < 0.001) (Figure 1).

**Figure 2:**
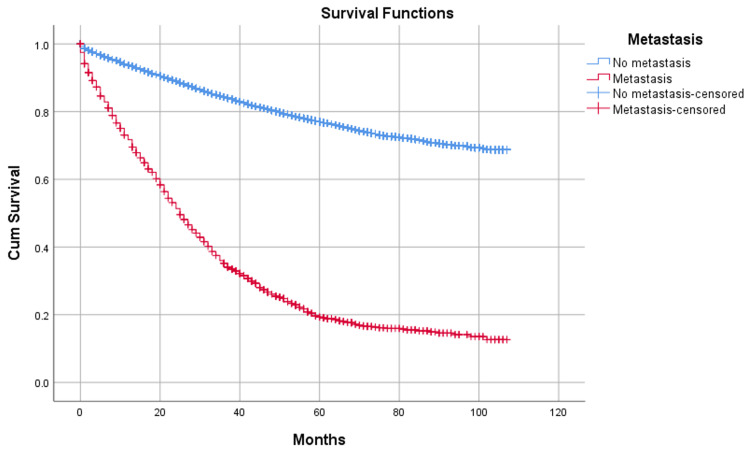
Median cancer-specific survival.

The median CSS of one, two, and three sites of metastasis were 29, 16, and 8 months, respectively (p < 0.001) (Figure 2).

We performed multivariate Cox regression to identify variables that influence OS and CSS in patients diagnosed with metastatic RAC, as demonstrated in Table 3. Variables significantly associated with decreased OS were age 50-75 years at diagnosis, age at diagnosis >75 years, grade 3 (high grade) tumors, T4 stage, N2 stage, tumors larger than 200 mm, no surgery, and multiple metastatic sites (p < 0.05). Variables significantly associated with decreased CSS were age >75 years, grade 3 (high-grade) tumors, T4 stage, N2 stage, tumors larger than 200 mm, no surgery, and multiple metastatic sites (p < 0.05).

**Table 3:** Multivariate Cox regression model for overall survival and cancer-specific survival in metastatic rectal adenocarcinoma.

Features	Overall survival	P-value	Cancer-specific survival	P-value
	aHR (95% CI)		aHR (95% CI)	
Race				
White	1 (Reference)		1 (Reference)	
Black	1.141 (0.954-1.364	0.148	1.177 (0.981-1.413)	0.08
Other	1.204 (1.010-1.435)	0.038	1.204 (1.004-1.443)	0.045
Gender				
Male	1 (Reference)		1 (Reference)	
Female	0.946 (0.844-1.061)	0.345	0.983 (0.874-1.106)	0.775
Age				
Less than 50	1 (Reference)		1 (Reference)	
50–75	1.154 (1.002-1.330)	0.047	1.097 (0.950-1.267)	0.207
More than 75	2.130 (1.786-2.540)	<0.001	2.009 (1.677-2.406)	<0.001
Tumor grade				
1	1 (Reference)		1 (Reference)	
2	1.116 (0.835-1.490)	0.459	1.112 (0.825-1.499)	0.486
3	1.924 (1.406-2.634)	<0.001	1.979 (1.431-2.735)	<0.001
4	1.436 (0.898-2.298)	0.131	1.487 (0.919-2.407)	0.106
T stage				
1	1 (Reference)		1 (Reference)	
2	0.930 (0.694-1.248)	0.63	0.862 (0.634-1.171)	0.341
3	0.885 (0.747-1.049)	0.16	0.852 (0.716-1.013)	0.07
4	1.310 (1.090-1.575)	0.004	1.278 (1.060-1.543)	0.01
Nodal status				
0	1 (Reference)		1 (Reference)	
1	0.946 (0.826-1.084)	0.426	0.965 (0.838-1.110)	0.614
2	1.257 (1.065-1.485)	0.007	1.304 (1.098-1.548)	0.002
Surgery				
No	1 (Reference)		1 (Reference)	
Yes	0.462 (0.404-0.529)	<0.001	0.450 (0.391-0.517)	<0.001
Tumor size				
<=200 mm	1 (Reference)		1 (Reference)	
200 mm	1.229 (1.069-1.413)	0.004	1.259 (1.092-1.453)	0.002
Income				
Less than 50,000	1 (Reference)		1 (Reference)	
50,000–75,000	0.926 (0.787-1.090)	0.356	0.937 (0.792-1.109)	0.452
More than 75,000	0.885 (0.742-1.055)	0.173	0.892 (0.744-1.071)	0.221

## Discussion

The metastatic spread in RAC patients complies with the widely accepted “anatomical/mechanical” and “seed-and-soil” hypotheses. The “seed” refers to tumor cells, and the “soil” refers to the organ microenvironment [15]. Recent advancements in investigating tumor-stroma interactions at the molecular level have refined the “soil-and-seed hypothesis.” Tumor cells have specialized characteristics at the molecular level that allow them to metastasize to specific target organs [16]. This hypothesis suggests that “metastasis at the first draining site acts as seeds to further metastasis.” The superior rectal vein carries the venous drainage from the proximal rectum, followed by the inferior mesenteric vein. The inferior mesenteric vein drains into the splenic vein, and the splenic vein then joins the superior mesenteric vein to form the portal vein, which ends in the sinusoidal network of the liver. This explains why the liver is the primary site of metastasis in RAC patients, particularly when the tumor originates in the proximal rectum. Blood then drains into the heart, from where it spreads into various other organs. The second most often sites metastasized are the lungs in proximal RAC patients. However, it becomes the first organ to be metastasized in patients who have developed adenocarcinoma in the distal rectum. Both colon cancer and RAC are frequently associated with liver metastasis; however, colon cancer patients presented with liver metastasis more often, and RAC patients presented with lung and bone metastasis more frequently [17]. During the routine evaluation of CRC patients, the brain is not usually imaged. Earlier detection of brain involvement in these patients has strong links to the timely selection of treatment and prevention. For example, the efficacy of stereotactic irradiation is higher if brain metastasis is detected earlier [17].

We analyzed several risk factors that were associated with metastasis. A correlation was noted using logistic regression among several risk factors, including gender, age, race, histological tumor grade, stage, surgical intervention, and socioeconomic status, to the incidence of metastasis. Sex difference is a major risk factor responsible for the distinct pattern of metastasis in RAC patients. Our findings support that the liver is the primary metastatic site for metastasis in RAC patients, followed by the lungs, bones, and brain. Moreover, liver metastasis was much more common in males compared to females. Manfredi et al. reported an increased incidence of liver metastasis in males compared to females [18]. However, Braunwarth et al. suggested that concerning the oncological outcome, there is no significant difference between the two sexes in terms of disease presentation and tumor metastasis, which contradicts our study [19]. Hence, deeper insight and research are recommended to study the relation of the differential metastatic spread between both genders.

Rarely, the brain becomes the metastatic organ in colon cancer or RAC. However, we noted the least number of patients in whom the tumor had metastasised to the brain, and most of these patients were women. A study suggested that increased levels of carcinoembryonic antigen and cancer antigen 19-9 and mutations in molecular markers, including *BRAF*, *NRAS*, and *KRAS*, are responsible for the involvement of the brain in RAC patients. Despite being rare, metastasis to the brain in RAC patients is frequently associated with lung metastasis, young age, and mutation [20]. The higher incidence of brain metastasis in female RAC patients is supported by Zhu et al. as well [21]. According to this study, *KRAS* mutations were more common in adenocarcinoma, while *BRAF* mutations were more frequent in female patients. Both os these factors, increased *BRAF* and *KRAF* mutations, are responsible for the more frequent involvement of the brain in women compared to men. Our findings are in line with the study of Gonsalves et al. and some other previous studies [22]. Chiang et al. studied the pattern of brain metastasis in patients with radically resected RAC. They reported the “incidence of brain metastasis after lung metastasis of 22.6%, of 3.6% after liver, and of 2.9% after local metastasis” [23]. However, some other studies claimed that there is no significant relation between *BRAF* mutation and gender, which contradicts our results [24].

According to our analysis and findings, age is an important risk factor for metastatic spread, with younger patients (<50 years) being particularly vulnerable to metastatic cancer. There is a strong correlation between age and the spread of distant organ metastasis that demands much more research to understand the metastatic disease pattern concerning age. Historically, as “cancer is often defined as a disease of ageing,” the incidence of rectal adenocarcinoma increases as a person ages. The aging process, despite being physiological, is responsible for the increase in the accumulation of mutations (deletions, point mutations, and translocations) that create a hyper-proliferative phenotype. Other contributing factors include epigenetic alteration, deregulation in nutrient sensing, telomere attrition, and impaired proteostasis; the age-related changes create a tumor-progressive environment [25]. A study on 797 patients, including young and aged patients with rectal cancer, suggested that there was no significant difference in the distant organ metastasis and clinical stage of the disease when comparing both age groups [26]. According to emerging data, age-related changes associated with the tumor microenvironment (e.g., inflammation), tumor biology (e.g., genomic instability), and the immune system (immunosenescence), along with the interaction between the genome and exosome, might have a significant contribution to the observed age-related metastatic pattern in RAC patients. As age is a significant prognostic factor, it should be a source of alarm for oncologists. Improvements in screening techniques and practices can contribute immensely to saving lives.

The literature on the relationship between metastatic patterns and race is relatively scarce. According to our results, African American patients had a higher incidence of developing any kind of metastasis, with liver and lung metastases being the most prevalent. Wan et al.’s findings are in line with our results, in which African American patients had 30% higher odds of developing liver metastasis and 24% higher odds for lung metastasis compared to Caucasians. In the African American population, a lack of access to routine screening, healthcare, and a low socioeconomic status may result in late diagnosis and a higher incidence of metastatic disease related to all cancers [27]. The association between the risk of distant metastasis and socioeconomic conditions was not found in rectal cancer patients belonging to the younger age group [28].

To our knowledge, this is the first SEER-based study providing deep insight into distant organ metastasis solely in RAC patients. With our findings regarding distant organ metastatic disease in RAC patients, we provided a valuable insight so that clinicians can make more informed decisions. Moreover, clinicians must consider rectal and colon cancers as separate entities. Keeping their differences in mind, they must design special and specific diagnostic, treatment, and follow-up plans. More research is required to provide a comprehensive understanding of the metastatic organs involved in RAC apart from CRC. Only then would we be able to explore new avenues of prevention and cure. Prevention and cure would stem from molecular, interventional, or prospective clinical studies.

This study has a few limitations. First, on the SEER database, a metastatic record of only four organs was available, including the liver, brain, bone, and lungs, leading to an underestimation of metastatic sites. However, metastasis in these four sites accounted for more than 90% of RAC patients, leaving other metastatic sites with a smaller proportion. Moreover, the SEER cancer registry does not update data routinely on recurrences and comorbidities, which might affect the treatment choice and survival. The availability of limited data on specific treatment modalities in addition to chemotherapy and surgeries has hindered us from studying treatment differences, which were important for racial disparities in survival.

## Conclusions

Among RAC patients, the liver was the most common organ to receive metastasis. Risk factors observed in RAC patients for metastasis were age <50 years, grade 4, T4, N2, and patients without surgery. Median OS in patients was different among patients with different numbers of metastatic sites, showing that OS decreases as the number of metastatic sites increases. Old age, multiple metastatic sites, high-grade tumors, T4, N2, tumors larger than 200 mm, and no surgery were strongly linked to a lower OS. The factors linked to a lower CSS were a high-grade tumor, multiple metastatic sites, no surgery, tumors larger than 200 mm, T4, N2, and old age. These findings may help identify high-risk groups that require close monitoring, as our research revealed site-specific metastatic risk factors.
